# Biological Evaluation of the Osteoinductive Potential of Dry Teeth after Chemical Demineralization Treatment Using the Tooth Transformer Device

**DOI:** 10.3390/biom13121727

**Published:** 2023-11-30

**Authors:** Sara Franceschelli, Rosalba Lagioia, Federica De Cecco, Elio Minetti, Andrea Ballini, Valeria Panella, Lorenza Speranza, Alfredo Grilli, Filiberto Mastrangelo

**Affiliations:** 1 Department of Medicine and Aging Sciences, University “G. d’Annunzio” Chieti-Pescara, Via dei Vestini 31, 66100 Chieti, Italy; sara.franceschelli@unich.it (S.F.); federica.dececco@unich.it (F.D.C.); valeria.panella@unich.it (V.P.); lorenza.speranza@unich.it (L.S.); alfredo.grilli@unich.it (A.G.); 2 UdA-TechLab, Research Center, University “G. d’Annunzio” Chieti-Pescara, Via dei Vestini 31, 66100 Chieti, Italy; 3 Department of Clinical and Experimental Medicine, University of Foggia, Via L. Rovelli n°48, 71122 Foggia, Italy; rosalba_lagioia.563488@unifg.it (R.L.); andrea.ballini@unifg.it (A.B.); 4 Department of Biomedical, Surgical, Dental Science, University of Milan, 20161 Milan, Italy; elio.minetti@unimi.it

**Keywords:** Tooth Transformer, osteoinductivity, Bone Morphogenetic Protein 2, mineralization protein LIM-1, transforming growth factor-β

## Abstract

Several studies have already demonstrated the biocompatibility of a tooth as a grafting material in the regeneration of bone tissue, showing its osteoconductive potential, while no studies have verified whether the osteoinductive potential of a tooth remains constant or is altered after its treatment with the Tooth Transformer (TT) device. The aim of the study was to demonstrate that the treatment with the TT device did not alter the osteoinductivity of an extracted tooth that was stored dry. Twelve extracted human teeth were collected from real patients. Caries, tartar and filling materials were removed from each tooth; each tooth was coarsely cut and stored at room temperature (RT) until use. Each sample was shredded, demineralized and disinfected, using the TT device. Protein extraction was carried out for each sample, and Western Blot analysis was performed to test the presence of mineralization protein LIM-1 and transforming growth factor-β. The presence of the human Bone Morphogenetic Protein 2 (BMP-2) and human collagen Type I (COL-I) was found in dry tooth samples processed with the TT device and subjected to Enzyme-Linked Immunosorbent Assay (ELISA) testing. The treatment of chemical demineralization using the TT device does not alter the osteoinductive potential of a dry tooth.

## 1. Introduction

Trauma, destructive caries, periodontal disease, osteolytic and neoplastic lesions are the most frequent causes of loss of dental elements, generating a partial or total edentulism of the maxillary bones, with a serious impact on social life and with a reduction in the quality of life due to damage to masticatory, phonatory and aesthetic functions [1]. In 1972, Tallgren conducted a mixed-longitudinal study on complete denture wearers. During the observation period (25 years), the resorption of the residual alveolar ridges caused a decrease in pre-extraction lower facial height (LFH). The reduction of the face height was mainly due to the resorption of the mandibular ridge and a consequent upward rotation and forward slide of the mandible [2]. In fact, the loss of one or more dental elements always involves a three-dimensional, vertical and horizontal bone resorption with changes in occlusal, musculoskeletal and articular ratios, and a slow aging of the patient’s face. This volumetric bone loss, which could compromise the correct rehabilitation of the maxillary bones, would seem to be linked to the loss of the functional masticatory load by the alveolar bone, which is an integral part of dental tissues and has the same mesenchymal embryonic origin [3].

In 1969, Johnson et al. showed that, after a tooth extraction, there is a resorption of alveolar bone ranging from 2.5 to 7 mm in height and up to 30 mm in width. This study also showed that most changes in the alveolar process occur during the first month after tooth extraction. There is a further, but less significant, bone resorption in the period from 10 to 20 weeks thereafter [4].

In 2003, Schropp et al. described, after 12 months of healing, a resorption of 50% at the level of a residual alveolar crest [5].

Cardaropoli et al. [6] and subsequently Araújo and Lindhe (2005) showed in an animal model how this three-dimensional bone resorption is not homogeneous but occurs more at the buccal maxillary bone (2.2 mm), while it is reduced at the palatal and lingual level (0.1 mm) [6,7].

In 2009, Van der Weijden et al. [8] published a systematic review regarding the changes in height and width of post-extraction sockets in humans. Their clinical data do not support the results as reported by Araújo and Lindhe (2005) [7]. In fact, the study outcomes showed that, during the post-extraction healing period, the amount of bone that is lost in width is greater than that lost in height. In particular, the clinical reduction in the width of the alveolar ridges was 3.87 mm, while the clinical bone loss in terms of height was 2.57 mm (1.67 mm was the vertical bone loss at the buccal aspect, while 2.03 mm was the vertical bone loss at the lingual aspect) [8].

Therefore, in the last ten years, numerous studies have evaluated the best surgical techniques and the ideal grafting material for the reconstruction of bone defects [9]. Furthermore, the clinical research results were absolutely predictable, and the results obtained for bone regeneration and repair by the means of heterologous or xenogenic bone grafts (obtained from cattle, pigs and horses) and synthetic biomaterials (hydroxyapatites, bioglasses, tricalcium phosphate, etc.) exhibited similar characteristics of biocompatibility, bio-inertia and porosity. Even today the autologous bone, taken from intraoral sites (chin symphysis, body and branch of the mandible) or extraoral sites (anterior iliac wing, tibia and parietal calvary), is considered the gold standard for its characteristics of biocompatibility, non-immunogenicity, osteoinductivity, osteoconductivity and osteogenicity [10,11,12,13,14].

However, there are also numerous limitations of intraoral and extraoral sampling linked to the different qualitative characteristics (ratio between cortical and medullary bone) and to the amount of tissue available. In addition to the possible intra- and post-operative complications, the other limitations are physiological resorption and the need for a second surgical site from which the bone tissue necessary for the three-dimensional reconstruction of the maxillary bones can be obtained [15]. 

In the last five years, a remarkable number of studies have been directed towards the study of minimally invasive surgical techniques and graft biomaterials to prevent or reduce the impact of resorption of the jaw bones; however, these biomaterials possess only osteoconductivity characteristics and reduce the regenerative potential [16].

In recent years, numerous clinical studies have used an extracted tooth to serve as a grafting biomaterial, for its characteristics of biocompatibility, non-immunogenicity, osteoinductivity, osteoconductivity and osteogenicity [17].

Numerous studies have evaluated how teeth and bones have similar chemical compositions, with dentin (D) exhibiting 70% inorganic component and 20% organic component compared to the alveolar bone (65% inorganic component and 25% organic component). In addition, both contain type I collagen (COL-I) (90%) and non-collagen proteins (10%), such as osteocalcin, osteonectin, sialoprotein and phosphoprotein, that are essential for the formation and mineralization of the bone matrix. Growth factors are also present, such as mineralization protein LIM-1 (LMP-1) and transforming growth factor-β (TGF-β) [18]. In 1998, Boden et al. demonstrated that LMP-1 is a positive regulator of osteoblast differentiation. In addition, LMP-1 can promote osteogenesis through the activation of the ERK kinase signaling pathway [19].

Bone Morphogenetic Proteins (BMPs) can induce the differentiation of mesenchymal stem cells into chondrocytes and promote bone neoformation [20].

In addition, TGF-β has been shown to collaborate with BMPs in inducing early osteoblast differentiation [21].

The inorganic component of D is represented by hydroxyapatite and three other biological calcium phosphates, i.e., tricalcium phosphate, octacalcium phosphate and amorphous calcium phosphate. These interact with each other, playing a positive role in bone remodeling. It has been observed by several researchers that this mineral component of D traps BMPs and limits their bioavailability; therefore, D’s demineralization is believed to promote the release of BMPs [22]. In a study conducted on naked mice, it was histologically confirmed that granules of fully demineralized human D can induce the independent formation of bone and cartilage; the demineralization seems to promote the release of growth factors and non-collagen proteins [23].

Since 1993, bone grafting materials have been produced from human teeth.

In 2008, autogenous tooth bone graft material (AutoBT) was introduced, i.e., a grafting material for the regeneration of bone defects, obtained from extracted teeth, prepared as a powder and grafted into the same donor patient. The organic component of AutoBT, containing Non-Collagenous Proteins (NCPs), has osteoinductive potential, while the inorganic component of AutoBT has osteoconductive potential. AutoBT can be used for sinus lift, guided bone regeneration (GBR), alveolar ridge augmentation and ridge preservation; the use of AutoBT has eliminated the risk of immune reactions [16].

In 2022, E. Minetti et al. showed, in a histological study, the excellent biocompatibility and high histomorphometric percentages of newly formed bones (38.0 ± 22.0%) at post-extraction sites treated with autologous tooth [24].

In addition, in 2021, E. Minetti et al. showed in a human study, after 1 year of follow-up, the high implant success rates (98.2%) at maxillary sites regenerated by autologous tooth after treatment with the Tooth Transformer (TT) device [25].

The TT device is an innovative patented device, which can transform an extracted dental element into a biocompatible autologous material for bone regeneration.

This medical device reduces the crystallinity of hydroxyapatite and makes available the morphogenetic proteins and growth factors present in D [26].

Several studies have already demonstrated the biocompatibility of a tooth as a grafting material in the regeneration of bone tissue, showing its osteoconductive potential. No studies have verified whether the osteoinductive potential of a tooth remains constant or is altered after their treatment with the TT device.

The aim of the present study is to demonstrate that an extracted tooth stored in dry a condition still has osteoinductive potential, after being processed with the TT device.

## 2. Materials and Methods

### 2.1. Sample Preparation

Twelve human teeth were extracted, after obtaining the signed informed consent forms from different patients. By the means of truncated cone diamond cutters for turbine and under constant irrigation with refrigerated sterile physiological solution, each tooth was cleaned of caries, tartar and filling materials, and then was roughly dissected and stored at room temperature (RT) until use. Each sample was weighed: the weight varied between 0.181 g and 1.396 g. Using the Tooth Transformer TT device (TT Tooth Transformer Srl Milan, Italy) and following the instructions provided by the manufacturer, each sample was shredded, demineralized and disinfected. The Tooth Transformer TT is commercially available; thus, an approval from the ethics committee was not required. In particular, the fragments of a tooth were placed in the Tooth Grinder, which is a container made of thermoplastic material and equipped with surgical steel blades that work at very low turns, to shred them to obtain particles with a diameter Ø < 1 mm; with a kit of disposable accessories, the particulate matter was demineralized, disinfected and rinsed. The granules are subjected to UVA rays and ultrasonic vibrations, with temperature variations that were always below 43° to avoid any damage to the proteins. At the end of the treatment (25 min), a basket contained the granulate, while a cylinder contained the exhausted liquids. The granules obtained were used for subsequent analyses.

### 2.2. Reagents

Demineralization reagent was provided by TT Tooth Transformer s.r.l. (Milan, Italy). The demineralization kit contained a cartridge with six liquids, i.e., two of these were liquids made up of hydrochloric acid (0.1 M) and H_2_O_2_ (10%), one was demineralized H_2_O and four were mineralized H_2_O necessary to remove, in four different phases of the process, the acid residues.

### 2.3. Western Blot Analysis

Briefly, following the treatment with TT, ≈50 mg of particles was transferred into 1.5 mL polypropylene tubes, and RIPA buffer was added to each sample. After incubation at 4 °C overnight and 3 freezing/thawing cycles, samples were sonicated by the means of a tip ultrasonicator, and protein was extracted. Total protein content was determined with BCA protein assay kit, according to the manufacturer’s instructions (Thermofisher, Monza, Italy). Western blot analysis was performed as described previously [27], using the following antibodies against Monoclonal Mouse LIM1 (R&D Systems, MAB2725; 1:1000), goat polyclonal TGF-β1 (R&D Systems, AF-246-NA; 1:300) and mouse monoclonal β-actin (Santa Cruz Biotechnology, Inc., Dallas, TX, USA). The blots were then incubated for 1 h at room temperature with goat anti-mouse secondary antibody (Sc-2005; 1:2000; Santa Cruz Biotechnology) or polyclonal goat anti-rabbit secondary antibody (Sc-66931; 1:5000; Santa Cruz Biotechnology). The nitrocellulose was scanned using a computerized densitometric system (Bio-Rad Gel Doc 1000, Milan, Italy). Protein levels were normalized to the protein content.

### 2.4. Measurement of BMP-2 and Collagen Type 1

The tissue levels of the human Bone Morphogenetic Protein 2 (BMP-2, CSB-E04507h) and human collagen type I (CSB-E13445h) were measured using the commercial ELISA kit (Cusabio (Houston, TX, USA), according to the instructions of the productor. Plates were scanned using a cooled, specialized Charge-Coupled Device. The integrated density values of the spots of known standards were used to generate a standard curve. Density values for unknown samples were determined using the standard curve for each analysis to calculate the real values in pg/mL. All steps were performed twice. The assay sensitivity was equal to 15.6 pg/mL. The intra- and inter-assay reproducibility was >90%. Duplicate values that differed from the mean by more than 10% were considered erroneous and therefore were repeated.

### 2.5. Statistical Analysis

Quantitative variables are summarized as the mean value and standard deviations (SD) in Tables. To assess the accuracy of fold change data, the 95% confidence interval (95% CI) and standard error (SE) were determined. A Student’s *t*-test for unpaired data was applied to evaluate the significance of differences. All tests were two-tailed. The threshold of statistical significance was set at *p* = 0.05. Data analysis was performed on GraphPad Prism 6 Software, version 6.01, 2012.

## 3. Results

### 3.1. Evaluation of Type I Collagen Content and BMP-2 Protein

**Figure 1:**
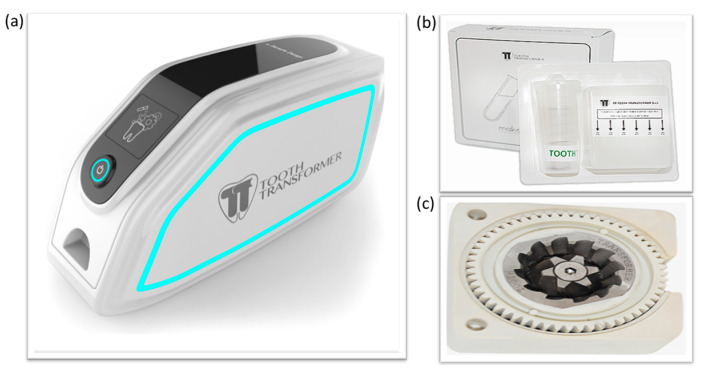
(**a**) The Tooth Transformer^®^, (**b**) tooth decontamination and demineralization kit, and (**c**) Tooth Grinder^®^.

Using the Enzyme-Linked Immunosorbent Assay (ELISA) test, the protein content of organic components, COL-I and BMP-2, was determined in dry tooth samples processed with the TT device (Figure 1).

The ELISA test showed that performing chemical demineralization with the TT device ensured the preservation of some osteoinductive proteins in the extracellular matrix (ECM) of a tooth, preserving the osteoinductive potential of dry tooth samples (Table 1).

### 3.2. Evaluation of Mineralization Protein LIM-1 (LMP-1) and Transforming Growth Factor-β (TGF-β)

**Figure 2:**
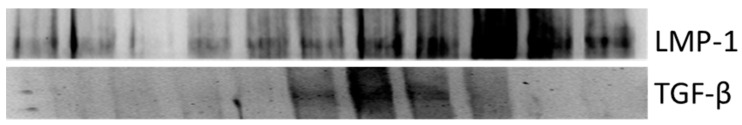
Analysis of mineralization protein LIM-1 (LMP-1) and transforming growth factor-β1 (TGF-β1) in tooth samples. Representative images of Western blot analysis of LMP-1 and TGF-β. Original images can be found in Figure S1.

In this study, the presence of LMP-1 protein and TGF-β was also evaluated in the analyzed samples. The mineralization protein LMP-1 is an intracellular osteogenetic factor, which produces an increase in the expression of some osteoinductive factors including BMP-2, as well as promotes the expression of TGF-β. In all analyzed samples, the presence of LMP-1 was demonstrated (Figure 2); however, the protein TGF-β does not follow the same trend.

## 4. Discussion

In recent years, several research groups have investigated the possibility of using extracted teeth, which are usually discarded as waste, as biocompatible autologous grafting material for the reconstruction of bone defects [16].

To date, the materials available for hard tissue regeneration are autologous bone, homologous bone, heterologous bone and synthetic biomaterials [28].

An ideal grafting material should have osteogenetic and osteoconductive potential, i.e., it can act as a scaffold for bone regeneration, and should have osteoinductive potential, i.e., it should favor the recruitment of cells that form new bone and induce the formation of the latter.

To date, autologous bone is still considered the reference material in bone regeneration procedures, as it is biocompatible, non-immunogenic and endowed with osteogenetic, osteoconductive and osteoinductive properties. The limitations of autologous bone are the reduced amount of material that can be collected from a donor site, as well as the morbidity and pain experienced at the donor site [13,15].

For several years, xenogenic bone, that is, a tissue derived from cattle, pigs or horses, has been successfully used in the field of reconstruction of bone defects of the jaws. Many studies have shown that heterologous bone is able to effectively act as a scaffold and to maintain space for the migration of osteogenetic cells. However, this material lacks osteoinductive properties. In fact, it has been found that the chemical or physical processes that are carried out to remove any organic residues from xenotransplanted materials cause the destruction of all osteoinductive proteins, which are essential in promoting bone regeneration [29].

Compared to xenotransplants, homologous bone grafts ensure faster bone turnover and, consequently, promote bone regeneration [30].

However, the materials mentioned above, which are currently commercially available for hard tissue reconstruction, have several limitations. These limitations mean that research in the field of bone regeneration must identify many more suitable grafting materials. In this scenario, the use of a tooth as a grafting material was considered since it has the same mesenchymal embryonic origin as the alveolar bone and a similar chemical composition [31]. D consists of a mineral component and an organic component, which consists mainly of collagen proteins and non-collagen proteins. In particular, the extracellular matrix (ECM) of D is made up of ≈90% COL-I. In small amounts, non-collagen proteins, such as osteocalcin, osteonectin and osteopontin, are also present, which play a key role in the mineralization of the collagen matrix [32]. It is also known that bioactive growth factors, such as TGF-β and BMPs, which are involved in bone tissue regeneration, are known to be present in a tooth. In fact, BMPs are essential in promoting osteodifferentiation and inducing bone formation; thus, they have been extensively studied [33,34]. However, BMPs can only be extracted from teeth in limited quantities. In this regard, studies have been conducted that have found that the reduced osteogenetic potential of a tooth can be attributed to the high amount of mineral component that, in some way, can trap BMPs, limiting their bioavailability. Therefore, it is believed that demineralization, through the reduction in the mineral component, can favor the release of these growth factors from a tooth [17]. In fact, several studies have concluded that the use of a demineralized tooth as a grafting material for the reconstruction of bone defects of the jaws is able to promote bone formation, through the preservation over time of autologous growth factors, such as osteopontin, sialoprotein, TGF-β and BMPs [35].

In 2021, Zhang et al. published a review article in order to compare AutoBT and other bone grafts. The experimental data showed that autogenous tooth grafts promote bone regeneration comparable to autogenous bone; the advantage of autogenous tooth grafts is that an additional surgery is not required to obtain a graft. Autogenous tooth materials are safer than allogeneic bone materials because they are obtained from the same individual. Compared with xenogeneic bone grafts, AutoBT showed an increased new bone formation; this could be due to the antigenicity of xenogeneic bone materials. Autogenous tooth grafts were more stable than the synthetic grafts; AutoBT performed better than synthetic bone materials in immediate implants [36].

Recently, a literature review selected 108 studies on autologous teeth used as graft material for bone regeneration, and finally considered six of them. The authors reported that the use of this innovative material in association with the placement of dental implants was associated with an implant survival rate of 97.7%. However, a frequent complication was surgical wound dehiscence [37].

In 2012, Reis-Filho et al. used human demineralized dentine matrix, obtained from extracted teeth, as bone graft material in tooth sockets of rats. This study demonstrated that human demineralized dentine matrix increased the expression of VEGF at days 7 and 14 and increased the formation of newly bone tissue in the sockets at 7, 14 and 21 days [38].

Another animal study showed that, compared to the use of polytetrafluoroethylene (PTFE) membrane alone, the use of PTFE membrane in combination with autologous demineralized dentine matrix (DDM) ensures a faster healing of treated bone defects [39].

Gomes et al. evaluated the effects of the autogenous demineralized dentin matrix on the dental socket wound healing process in humans, using the guided bone regeneration technique with a PTFE barrier. The radiographic bone density of the newly formed bone in the dental sockets was measured through densitometric analysis. During the 15-day and 30-day periods of healing, the radiographic analysis showed that autogenous demineralized dentin matrix promotes the formation of a homogenous and uniform trabecular bone. On the 90th day of healing, the radiographic bone density of the dental sockets treated with autogenous demineralized dentin matrix was similar to that of the surrounding normal bone [40].

Pang et al. made a comparison, considering 33 clinical cases of ridge preservation after tooth extraction, between clinical and histological performances of inorganic bovine bone and clinical and histological performances of autologous DDM obtained from the extracted tooth. In both cases, new bone formation and vertical bone augmentation were found, without any statistically significant difference [41].

Kim YK et al. carried out a study on 15 patients undergoing GBR, using an autologous tooth as a grafting material. These patients were followed-up for a period of 31 months. These authors concluded that a tooth has osteoconductive potential, and in fact, it produces a favorable bone healing [42].

A pilot study was conducted by Minamizato et al. in order to examine the efficacy and safety of autogenous partially demineralized dentin matrix (APDDM) in bone regeneration procedures related to implant dentistry, including socket preservation, maxillary sinus floor augmentation, and alveolar ridge augmentation. In the same tooth extraction session, APDDM was transplanted into the defect, and the implant was placed. In all cases, the graft sites healed without any notable complications, and oral rehabilitation using dental implants was successful for at least 2 years. The histological examination showed that APDDM was surrounded by newly formed bone. The advantage of APDDM is the short preparation time due to partial demineralization [43].

Recently, a study has been published on the different devices on the market that can make a tooth a suitable material for grafting in a bone defect. Such devices are the BonMaker, the TT and the Smart Dentin Grinder [44].

However, the present study was conducted focusing on the use of the Tooth Transformer to treat dry teeth. Over the years, several studies have shown that a tooth has osteoconductive potential. This study was conducted to demonstrate, for the first time, that a tooth also has osteoinductive potential and that it could, consequently, represent an ideal substitute for autologous bone, which, to date, still represents the reference material in the field of oral bone regeneration. In this study, the extracted dry teeth were processed with the Tooth Transformer device to demonstrate the content of osteoinductive factors. All samples revealed the presence of COL-I and BMP-2, providing convincing evidence that the treatment of a tooth with the Tooth Transformer device does not damage the ECM of the analyzed samples.

Contrary to previous studies, according to which demineralization could cause a severe depletion of BMPs in bone tissue, the present study has shown that the demineralization of a dry tooth, obtained by treating it with the Tooth Transformer device, guarantees the preservation of the structural and functional proteins of the ECM [45]. In the analyzed samples, the presence of the intracellular mineralization protein LMP-1 was also found. LMP-1 plays a key role in bone regeneration. In fact, it increases the bioavailability of BMP-2 and, consequently, enhances the osteoinductive capacity of the grafting material obtained from a tooth [19].

In 2012, Long Xiong et al. [46] showed the osteoinductive capability in novel hollow hydroxyapatite microspheres with Bone Morphogenetic Protein 2 (BMP2), as a potent osteogenic inducer of bone regeneration of large bone defects in rabbit model.

In 2019, Bin Ren et al. [47], in the bone tissue engineering sites of fascia layer with BMP-2, have shown a significant upregulation of bone marker genes, proteins and calcium deposition in an animal preclinical study.

As reported by Pan H et al., the LMP-1 regulation of BMP-2 occurs at the transcriptional level through the modulation of Runt-related transcription factor 2 (Runx2) [48]. Runx2 is a member of the runt domain family and plays crucial roles in osteogenic differentiation and maturation [49]. The activation of Runx2 in osteogenic precursor cells, during osteoblast differentiation and maturation, causes the expression of osteoblastic markers, such as COL1 and osteocalcin (OCN). The overexpression of Runx2 significantly increased BMP-2 promoter activity with LMP-1. At the molecular level, ERK1/2 MAPK activation is critical for the LMP-1-induced upregulation of the transactivity of the transcription factor Runx2 and the subsequent induction of BMP-2 [48]. This crosstalk between BMP-2 and Runx2, activated by LMP-1, plays a crucial role during the process of osteogenic differentiation and mineralization.

## 5. Conclusions

The present study demonstrated that treatment of chemical demineralization using the Tooth Transformer device did not alter the osteoinductive potential of an extracted tooth stored in a dry condition. All samples revealed the presence of COL-1 and BMP-2, providing convincing evidence that a treatment with the Tooth Transformer device does not damage the ECM of a tooth. Furthermore, LMP-1 was found in all analyzed samples. For the first time, the experimental data showed that a tooth not only has osteoconductive potential but also has osteoinductive potential. In the field of research looking into the ideal grafting material for the reconstruction of bone defects, an extracted tooth could be used in clinical practice to prevent or reduce the bone resorption following tooth loss. To date, an extracted tooth is discarded as waste; however, its characteristics of biocompatibility, non-immunogenicity, osteoinductivity and osteoconductivity suggest the possibility of it being used for the regeneration of bone defects, which have a significant impact on the patient’s function and aesthetics. The aim of our research group is to improve the experimental data, so that a tooth can be used in clinical practice as an autologous bone grafting material.

## Figures and Tables

**Table 1:** Content of COL-1 and BMP-2 in tooth samples.

Title 1	pg/mL
COL-I	3.64 ± 1.78
BMP-2	0.60 ± 0.48

COL-I: type I collagen (COL-I); BMP-2: Bone Morphogenetic Protein-2.

## Data Availability

No new data were created.

## Supplementary Materials

The following supporting information can be downloaded at: https://www.mdpi.com/article/10.3390/biom13121727/s1, Figure S1: Original images of Figure 2.

## Abbreviations

TT: Tooth Transformer; D: dentin; BMP-2: Bone Morphogenetic Protein 2; COL-I: human collagen type I; ELISA: Enzyme-Linked Immunosorbent Assay; LMP-1: mineralization protein LIM-1; TGF-β: transforming growth factor-β; NCPs: Non-Collagenous Proteins; GBR: guided bone regeneration; AutoBT: autogenous tooth bone graft material; ECM: extracellular matrix; DDM: demineralized dentine matrix; PTFE: polytetrafluoroethylene; LFH: lower facial height; APDDM: autogenous partially demineralized dentin matrix; OCN: osteocalcin; and Runx2: Runt-related transcription factor 2.
