# Supplementary figures and images for: Biological Evaluation of the Osteoinductive Potential of Dry Teeth after Chemical Demineralization Treatment Using the Tooth Transformer Device
Source: Biomolecules. 2023 Nov 30;13(12):1727. doi: 10.3390/biom13121727 (PMC10741675; doi:10.3390/biom13121727)

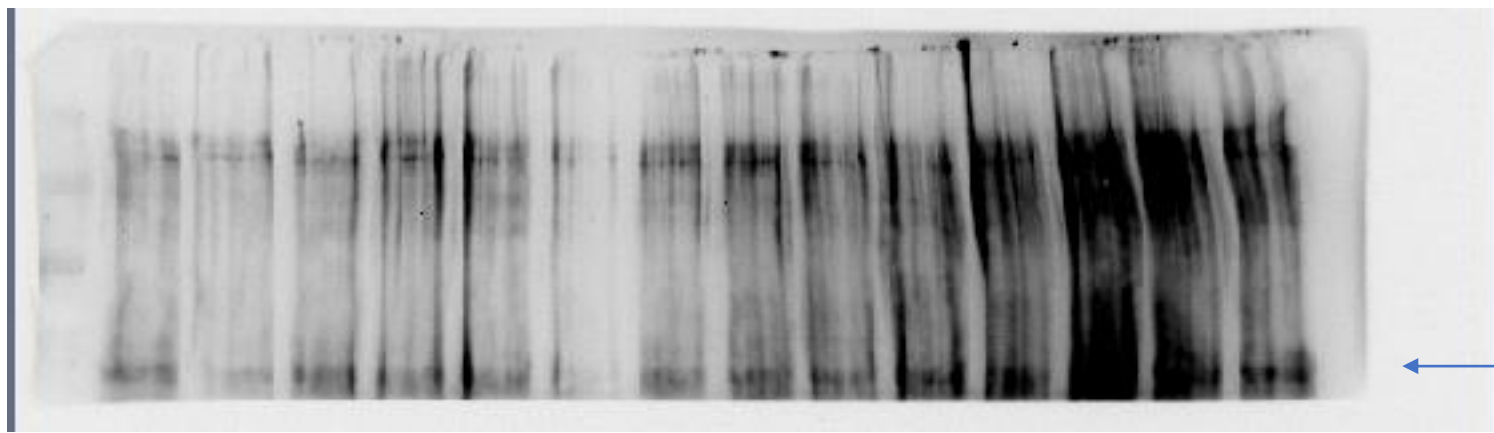

LMP 1

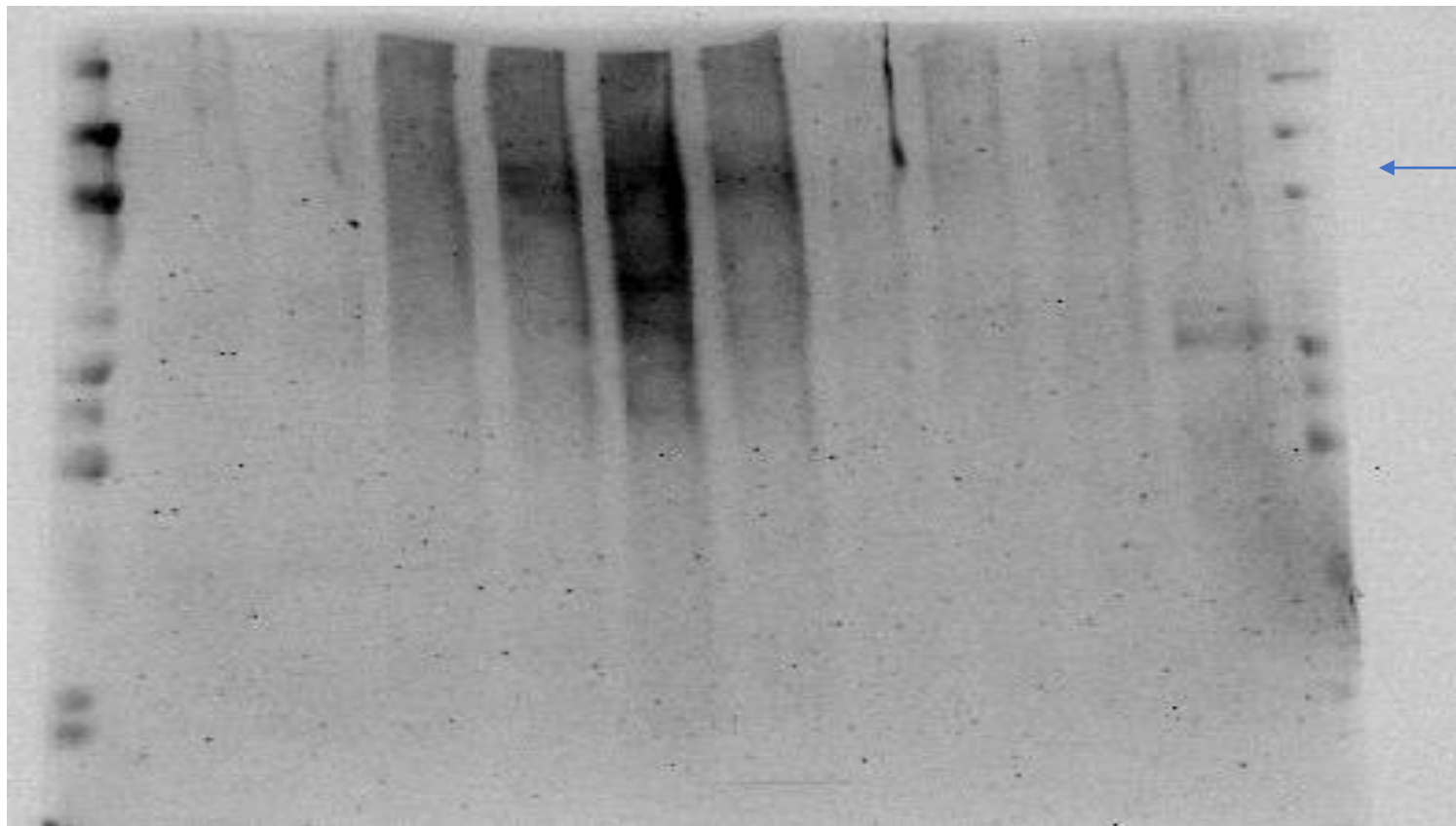

TGF- $\beta$

Figure S1: Original images of Figure 2.

Supplement: Supplementary file 1 [file biomolecules-13-01727-s001.zip › biomolecules-2680388-supplementary.pdf]
